# Live Combined *Bacillus subtilis* and *Enterococcus faecium* Ameliorate Murine Experimental Colitis by Immunosuppression

**DOI:** 10.1155/2014/878054

**Published:** 2014-09-08

**Authors:** S. Chen, Y. Fu, L. L. Liu, W. Gao, Y. L. Liu, S. H. Fei, Y. Tan, K. F. Zou

**Affiliations:** ^1^Cancer Center, Union Hospital, Tongji Medical College, Huazhong University of Science and Technology, Wuhan 430022, China; ^2^Department of Gastroenterology, Union Hospital, Tongji Medical College, Huazhong University of Science and Technology, Wuhan 430022, China; ^3^Division of Nephrology, Tongji Medical College, Huazhong University of Science and Technology, Wuhan 430030, China; ^4^Department of Traumatic Surgery, Tongji Medical College, Huazhong University of Science and Technology, Wuhan 430030, China; ^5^Department of Rehabilitation, Tongji Medical College, Huazhong University of Science and Technology, Wuhan 430030, China

## Abstract

Live combined *Bacillus subtilis* and *Enterococcus faecium* ameliorate murine experimental colitis by immunosuppression manifested by downregulation of TLRs, macrophages, Th1, and Th2 but upregulation of Tregs.

## 1. Introduction

Inflammatory bowel disease (IBD) is an idiopathic disorder of chronic inflammation of the gastrointestinal tract, which is represented mainly by ulcerative colitis and Crohn's disease. Despite scientific efforts during the last decades, etiology and pathogenesis of the two major inflammatory bowel diseases, namely, Crohn's disease and ulcerative colitis, remain rather unclear [1, 2]. The widely accepted causes are infections and immune and genetic factors. More and more recent reports have shown the importance of immune modulation in the development of IBD [3, 4]. Aminosalicylic acid, glucocorticoid, and immunosuppressant are three main therapies [5]. Recently some kinds of probiotics have been applied and shown to be significantly effective to IBD [6]. Since 2008, probiotic lactobacillus strains have been used to treat IBD as “B-grade” advice [7]. Probiotics are a group of specific nonpathogenic bacteria that are functionally and genetically defined by their ability to reduce inflammation in the intestine [8]. The mechanism is still under investigation. Recent studies relative to the mechanism of action of probiotics have shown that these organisms can have an effect on enhancing epithelial barrier function and modulating epithelial cytokine secretion into an anti-inflammatory dominant profile [9–11]. Our study aims to further investigate the effect of the probiotic lactobacillus strains Medilac-S (live combined* Bacillus subtilis* and* Enterococcus faecium* enteric-coated capsules) in murine experimental colitis and its possible mechanisms.

## 2. Materials and Methods

### 2.1. Animal

Male and female Sprague-Dawley rats (200–230 g) were obtained from the Tongji Laboratory Animal Center (Wuhan, China).

### 2.2. Preparation of Murine Model of Colitis

The murine models of colitis were randomly divided into three groups: normal group, control group, and Medilac-S group. Every group had 10 rats in the end. Experimental murine colitis models were established as follows [12]: fast 24 h, intraperitoneal anesthesia with pentobarbital (30 mg/kg), enema with 2,4,6-trinitrobenzene sulfonic acid (TNBS, 100 mg/kg, Sigma company), and equal volume anhydrous ethanol. After the establishment of experimental colitis, the saline (2 mL) and Medilac-S (20 mg/200 g, each 250 mg capsule contains 5.0*e* + 07* Bacillus subtilis* and 4.5*e* + 08* Enterococcus faecium*, Beijing Han Mei Pharmaceutical Co., LTD.) were administered into control group and Medilac-S groupby daily gastric irrigation. All rats were sacrificed after 10 days. All procedures were performed in accordance with the guidelines for animal care from the Animal Ethics Committee of Tongji Medical College, Huazhong University of Science and Technology.

### 2.3. Determination of DAI, CMDI, and Histological Change

Body weight, diarrhea, and bloody stool with naked eye were observed every day from the second day of establishment of the colitis model, which were scored as disease activity index (DAI): score 0 = weight without falling, stool normal, or occult blood (−); score 1 = weight loss 0–5%, loose stool, or occult blood (+); score 2 = weight loss 5–10%; score 3 = weight loss 10–15%, watery stools, or bloody stool with naked eye; score 4 = weight loss > 15; DAI = (weight loss score + stool score + hematochezia score)/3. After the sacrifice of the rats, the intestinal segments from the anus to ileocecal junction were taken and cut along the longitudinal mesenteric edge. The colon general state and colonic mucosa damage index (CMDI) [3] were evaluated by the anatomical microscope. The distal colon, transverse colon, and ascending colon were given a block size about 2 mm × 10 mm tissue specimens and in serious inflammation or ulcer place at least a piece of tissue sample was taken from each rat. Routine paraffin section, H&E staining, and pathology scores were performed by pathology professionals.

### 2.4. Determination of SOD and MPO Activities

SOD activity of tissue was analyzed by xanthine oxidase method and MPO activity of tissue was measured by spectrophotometry according to the specific steps for SOD, MPO detection kits (Nanjing Jiancheng Biological Engineering Research Institute).

### 2.5. Reverse Transcription Quantitative Real-Time PCR

RNA was extracted using the Trizol method (Invitrogen, UK) according to the manufacturer's instructions. A volume of 1 mg of RNA was reverse transcribed using SuperScript II reverse transcriptase and Oligo (dT) primers (Invitrogen, UK). The mRNA expressions of TLR2, TLR4, and TLR9 were quantified using SYBR green master mix (Finnzyme, New England Biolabs, UK) and *β*-actin for normalization among samples. The primers used are listed as below: TLR2 upstream primer 5′AAACGGTAACAATACGGAG3′, downstream primer 5′ TGACAACTGTC GGGCATA3′; TLR4 upstream primer 5′CAGAGCCGTTGGTGTATC3′, downstream primer 5′CCCTGTGAGGTCGTTGA3′; and TLR9 upstream primer 5′AGTGCTTGATGTGGGTGG3′, downstream primer 5′ CTGAGCGTGTTC TTGTTGA3′. PCRs were performed as 40 cycles of 95°C (15 s), 60°C (45 s), and 72°C (45 s) on a Chromo 4 cycler and recorded with MJ Opticon Monitor software V3 (Biorad Labs Inc., Hercules, CA, USA). Gene expression was calculated relative to *β*-actin.

### 2.6. Immunohistochemistry

3 *μ*m thick paraffin sections were in sequence incubated with 3% H_2_O_2_ for 10 min and 5% BSA for 30 min at room temperature and then incubated with primary antibodies which are rabbit anti-rat and polyclonal such as TLR2 (sc - 10739), TLR4 (sc - 10741), and TLR9 (sc - 25468).

The paraffin sections were then incubated with biotinylated secondary goat anti-rabbit IgG for 30 min followed by horse radish peroxidase-conjugated streptavidin (BD Pharmingen, USA) and diaminobenzidine staining; an irrelevant rabbit IgG was used for negative control. Images from the microscope were captured with a Nikon DXM 1200 digital camera using Automatic Camera Tamer (ACT-1) software and analyzed with image analysis software (Image Pro plus 5.1). The expressions of TLRs were quantified by the mean numbers of positive cells per square millimeter at 400x magnification on 5 fields per section from 10 rats per group. All scorings were carried out by observers blinded to the experimental groups.

### 2.7. Immunofluorescence

The tissue sections (6 um) were immunostained with the primary antibody, rabbit anti-rat F4-80 (1 : 100; Santa Cruz Biotechnology), PE-conjugated anti-rabbit secondary antibody (1 : 200; Invitrogen), and 40,6-diamidino-2-phenylindole (DAPI, Invitrogen). Images were analyzed using a BIOREVO immunofluorescence microscope (Keyence). Each result was obtained in at least four separate experiments. We prepared ten rats in each group for a single experiment.

### 2.8. Flow Cytometry

Spleens were harvested and single cell suspensions were prepared by processing the spleen with a 200 mm nylon mesh. The spleen cells were directly collected in a 35 mm dish which was filled with 4 mL EZ-Sep mouse 1X lymphocyte separation medium (Dakewe Biotech Company Ltd., Shenzhen, China). Then the cell suspension in lymphocyte separation medium was transferred into a 15 mL centrifuge tube. RPMI 1640 medium (1 mL) was laid on it. The tube was centrifuged at 800 g for 30 min at 4°C. Red blood cells and dead cells were deposited at the bottom. Lymphocytes at the interface were collected and washed.

The lymphocytes were labeled with anti-CD4-FITC (Biolegend, CA, USA) and anti-CD25-PE (eBioscience, CA, USA) monoclonal antibodies. After staining with CD4-FITC, the membranes of cells were fixed and ruptured and then the cells were stained with anti-intracellular cytokines monoclonal antibodies such as anti-IFN-*γ*-PE (eBioscience, CA, USA) or anti-IL-4-PE (eBioscience, CA, USA). Samples were acquired using a FACScan cytometer (Beckton Dickinson, Oxford, UK) and analyzed with “FCS Express V3” software (De Novo Software).

### 2.9. Statistical Analysis

Data are expressed as mean ± SD. Statistical differences were analyzed by Student's *t*-test or a one-way ANOVA using SPSS (version 13.0). A *P* value < 0.01 was considered statistically significant.

## 3. Results

### 3.1. Medilac-S Ameliorated Murine Experimental Colitis

**Figure 1:**
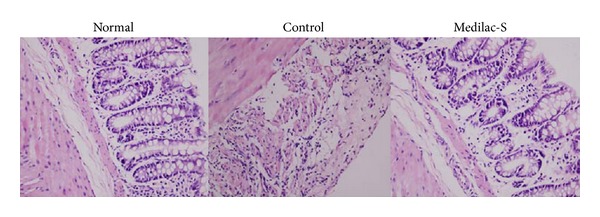
Morphological analysis of the lower segments of colons from normal, control, and Medilac-S rats. H&E staining with a magnified view ×200 shows significant histological changes in groups. In the control group, the glands were destroyed to a certain extent. An amount of neutrophils and lymphocytes was seen in intestinal tissue and a few eosinophils infiltrated into the intrinsic layer and submucosa. But in the Medilac-S group, few inflammation cells were seen (bars = 200 *μ*m).

Congestion, edema, erosion and bleeding, and shallow small ulceration were indicated in the intestinal tissues of experimental colitis rats, particularly in lower segments of colons. The glands were destroyed to a certain extent. An amount of neutrophils and lymphocytes was seen in intestinal tissue and a few eosinophils infiltrated into the intrinsic layer and submucosa, which could be accompanied with crypt abscesses, granuloma, and thickening of mucosa muscle layer (Figure 1). Histologic change, DAI, and CDMI scores of control group were significantly higher compared with normal group (*P* < 0.01) (Table 1). After treatment with Medilac-S, the pathologic changes in experimental colitis rats were ameliorated (Figure 1). Histologic change, DAI, and CDMI scores of Medilac-S group were significantly reduced compared with control group (*P* < 0.01) (Table 1).

The activities of SOD were lower (*P* < 0.01) and the activities of MPO were higher (*P* < 0.01) in control group compared with normal group (Table 2). After treatment with Medilac-S, the activities of SOD were significantly increased (*P* < 0.01) and the activities of MPO were decreased (*P* < 0.01) in experimental colitis rats (Table 2).

### 3.2. Medilac-S Reduced the Expression of TLRs in Experimental Colitis Rats

**Figure 2:**
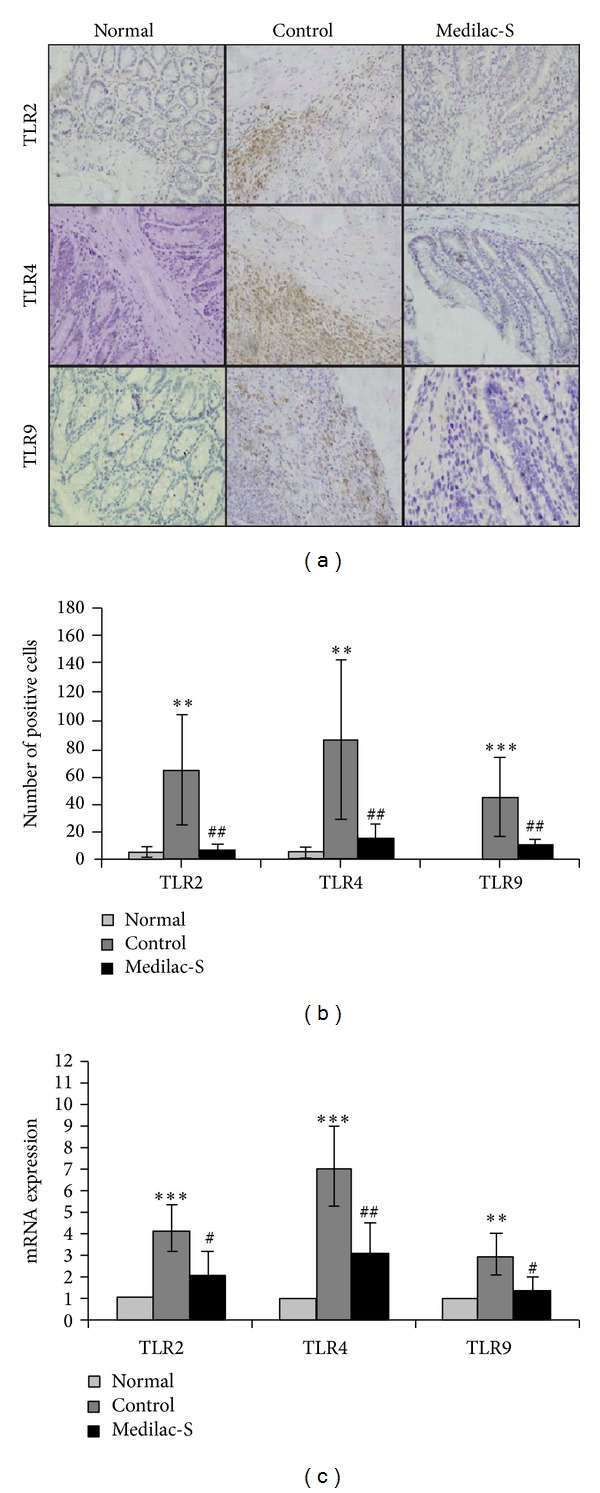
The expressions of TLRs in groups. (a) Immunochemistry of TLRs expressions in groups. (b) The mean numbers of positive cells per square millimeter of TLRs expressions were quantificated. (c) qRT-PCR analyzed the mRNA expressions of TLRs in groups. TLR2 and TLR4 were lowly expressed in normal intestinal tissues, while TLR9 was rarely expressed. After the establishment of experimental colitis, the expressions of TLR2, TLR4, and TLR9 were enhanced in the inflammatory cells which were located in lamina propria and submucosa. But after the treatment of Medilac-S the expressions of TLR2, TLR4, and TLR9 were all weakened. And the mRNA expressions in groups had the similar phenomenon (*n* = 10 per group; ∗compared with normal group, ^#^compared with control group, ∗ or ^#^ *P* < 0.05, ∗∗ or ^##^ *P* < 0.01 and ∗∗∗ or ^###^ *P* < 0.001).

In the normal group TLR2 and TLR4 were lowly expressed in the intestinal tissues, while TLR9 was rarely expressed. After the establishment of experimental colitis, the expressions of TLR2, TLR4, and TLR9 were enhanced in the inflammatory cells which were located in lamina propria and submucosa (Figure 2(a)). But after the treatment of Medilac-S the expressions of TLR2, TLR4, and TLR9 were very weak (Figures 2(a) and 2(b)). In accordance with the above results, the mRNA expressions of TLRs in control group were higher compared with the normal group, but after treatment with Medilac-S the mRNA expressions of TLRs reduced (Figure 2(c)).

### 3.3. Medilac-S Reduced the Infiltration of Macrophages in Experimental Colitis Rats

**Figure 3:**
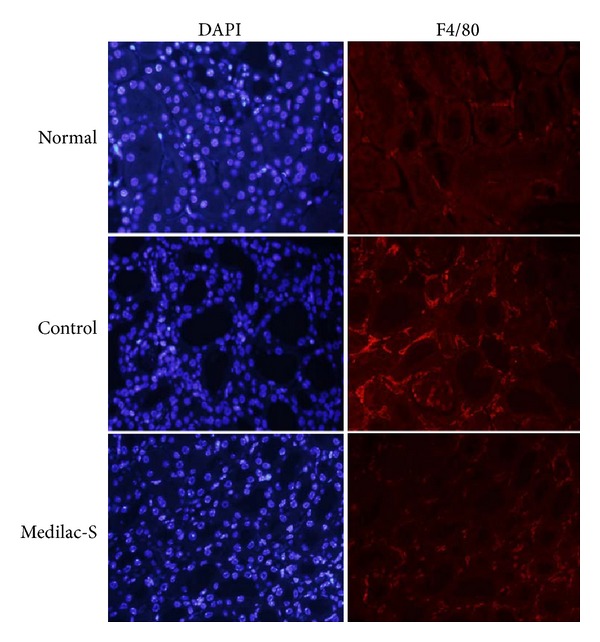
Infiltration of macrophages into the lower segments of colons from normal, control, and Medilac-S rats. Representative images of lower segments of colons tissue stained with DAPI (blue) and F4/80 (red). The result has shown that rare macrophages labeled with F4/80 infiltrated in the intestinal tissue in the normal group. But after establishment of murine model of colitis, a few macrophages infiltrated into the local intestinal. And the treatment with Medilac-S reduced the infiltrated macrophages significantly.

The infiltration of macrophages in the local intestinal tissue is an important early event during the process of colitis [13], which could recruit other inflammatory cells into local tissue. So we compared the states of the infiltration of macrophages in the three groups. The result has shown that rare macrophages infiltrated into the intestinal tissue in the normal group. But after establishment of experimental colitis, more macrophages labeled with F4/80 infiltrated into the local intestinal. By the treatment with Medilac-S to control group, the infiltrated macrophages reduced significantly (Figure 3). These results imply that Medilac-S could prevent the infiltration of macrophages into local intestinal tissues.

### 3.4. Medilac-S Decreased the Percentage of Th1 and Th2 but Increased the Percentages of Tregs in Experimental Colitis Rats

**Figure 4:**
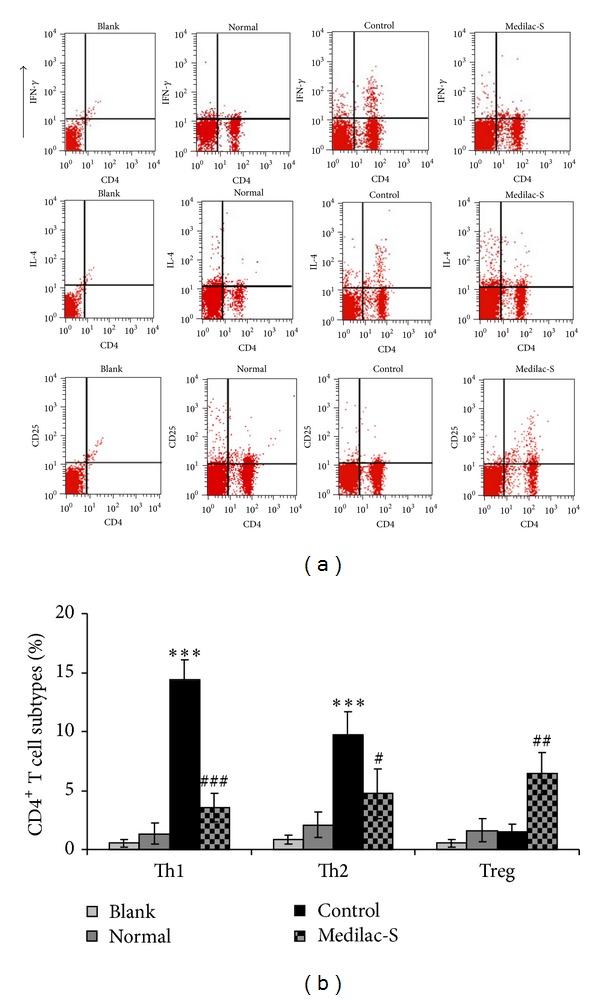
Analysis of subtypes of CD4^+^ T lymphocytes from the spleens of rats in groups. (a) FACS analysis of Th1 (CD4^+^ IFN-*γ* ^+^), Th2 (CD4^+^ IL-4^+^), and Treg (CD4^+^ CD25^+^). (b) Quantification of the percentages of Th1, Th2, and Treg in lymphocytes of the spleen. Our results demonstrated that significant higher activation of Th1 and Th2 in control group compared with the normal group. Medilac-S treatment decreased the ratios of Th1 and Th2 but increased the ratio of Tregs significantly compared with the control group. (*n* = 10 per group; ∗compared with normal group, ^#^compared with control group, ∗ or ^#^ *P* < 0.05, ∗∗ or ^##^ *P* < 0.01, and ∗∗∗ or ^###^ *P* < 0.001).

CD4^+^ T lymphocytes activation is a crucial immune response accompanied by inflammation and the ratios of subtypes of CD4+ T lymphocytes such as Th1, Th2, and Tregs manifest the state of immune system. We aimed to analyze the ratios of subtypes of CD4^+^ T lymphocytes in the three groups and find the effect of Medilac-S on the state of immune response. Our results demonstrated that significant higher activation of Th1 (*P* < 0.001) and Th2 (*P* < 0.001) in control group compared with the normal group which imply excessive and continuous inflammation and immune response (Figures 4(a) and 4(b)). Medilac-S treatment decreases the ratios of Th1 (*P* < 0.001) and Th2 (*P* < 0.01) but increases the ratio of Tregs (*P* < 0.005) significantly (Figures 4(a) and 4(b)). These data indicated that Medilac-S has a role of immunosuppression manifested by downregulation of Th1 and Th2 and upregulation of Tregs.

For a more complete evaluation, all experiments consisting of rats model received heat-treated Medilac-S (121°C, 15 min in high compressed steam). Interestingly, the observed effects of live Medilac-S were all abolished (data not shown). So the heat-treatment destroys the probiotic character of Medilac-S.

## 4. Discussions

Inflammatory bowel diseases (IBD) are chronic, relapsing inflammatory disorders of the gastrointestinal tract. Although the cause of IBD remains unknown, immunological abnormalities triggered by genetic and environmental factors are thought to be important in its pathogenesis [2].

The majority of patients with IBD were used conventional therapy (viz., aminosalicylates, antibiotics, corticosteroids, and immunomodulatory agents) to both induce and maintain remission [14]. There is mounting evidence that probiotic therapy may ameliorate IBD both in animal models and in patients [8]. Probiotics, for the treatment of IBD, are a group of specific nonpathogenic bacteria that are functionally and genetically defined by their ability to reduce inflammation in the intestine. Although probiotics also seem to have broad beneficial effects in humans, there are specific identified mechanisms for the pathogenesis of IBD. Probiotics have been reported to have a direct effect on epithelial cell function and intestinal health, including enhancing epithelial barrier function, modulating epithelial cytokine secretion into an anti-inflammatory dominant profile, and altering mucus production [15, 16]. But the effect of probiotics on the innate or adaptive immune in IBD remains unclear since immunological abnormalities are important in its pathogenesis.

The group chose Medilac-S (live combined* Bacillus subtilis* and* Enterococcus faecium* enteric-coated capsules) to study the probiotic effect and mechanism. The* Bacillus subtilis* species has a long history of safe use. In view of the fact, some strains of* Enterococcus* can display a high level of resistance to vancomycin or can acquire such resistance and that certain strains of vancomycin resistant enterococci are commonly associated with nosocomial infections in hospitals. Meanwhile, some strains of* Enterococcus* display probiotic properties and may not at the point of inclusion in a product display vancomycin resistance. So we choose normal gut bacteria* Enterococcus faecium*, which is of course vancomycin sensitive.

TLRs represent key mediators of innate host defense in the intestine, involved in maintaining mucosal as well as commensal homeostasis [17]. The TLR family includes cellular signatures (mainly TLR2, TLR4, and TLR9) of microbial pathogens and plays a fundamental role in innate immune responses. The signal transduction is initiated from the Toll/interleukin-1 receptor (TIR) domain of TLRs after pathogen recognition. Almost all TLRs use a TIR-containing adapter MyD88 to activate a common signaling pathway that results in the activation of NF-kappaB to express cytokine genes relevant to inflammation leading to the production of inflammatory cytokines, chemokines, and interferons [18]. But inappropriate natural immune response induced by TLR4 may lead to serious tissue organ damage. Undue TLR stimulation may disrupt the fine balance between pro- and anti-inflammatory responses. Such disruptions may harm the host through the development of autoimmune and inflammatory diseases, such as rheumatoid arthritis and systemic lupus erythematosus [19]. Our previous study has shown that high expressions of TLR2, TLR4, and TLR9 in the colonic mucosa of experimental colitis rats and a positive correlation between intestinal damage degrees, which implied TLR2, TLR4, and TLR9, prompt the immune injury of murine intestinal in experiment colitis [20]. In this study, our results have shown that Medilac-S alleviated the protein and mRNA expressions of TLR2, TLR4, and TLR9 in the colon mucosa in experimental colitis. We assume that Medilac-S ameliorated murine experimental colitis by downregulation of TLRs which would suppress inflammation and innate immune response.

Our results also show that fewer macrophages were infiltrated after Medilac-S treatment in experimental colitis. Reports have shown that TLRs could induce neutrophil recruitment in lungs [21]. Medilac-S may decrease the recruitment of macrophages in intestinal by suppression of chemokines induced by TLRs, which would be further investigated.

TLRs also have crucial roles in initiating and shaping the adaptive immune response. Our results also have shown that the percentages of Th1 and Th2 cells were decreased but the percentage of Tregs was increased after Medilac-S treatment in experimental colitis. It seems that Medilac-S could play a role in the immune shift from T helper cells to Tregs. Recent researches have shown that TLRs could regulate the activation of T cells. TLRs also play an important, indirect role in the initiation of subsequent adaptive T cell responses. TLRs can function as costimulatory receptors that complement TCR-induced signals to enhance effector T cell proliferation, survival, and cytokine production [22–24]. But whether Medilac-S regulates T cell response via TLRs is still uncertain.

In conclusion, Medilac-S has protective effect against murine experimental colitis with decreased expression of TLRs, less infiltration of macrophages, and regulation of T subtypes. The accurate signaling mediated by Medilac-S needs to be further investigated.

## Figures and Tables

**Table 1:** DAI, CDMI, and histological change in groups.

Groups	Numbers	DAI	CDMI	Histological change
Normal	10	0.07 ± 0.14	0.00 ± 0.00	0.20 ± 0.42
Control	10	1.72 ± 0.58**	8.45 ± 0.51**	6.54 ± 0.51**
Medilac-S	10	0.60 ± 0.78^##^	1.20 ± 1.40^##^	1.45 ± 1.01^##^

Note: **P < 0.01 compared with normal group; ^##^ *P* < 0.01 compared with control group.

**Table 2:** The activities of SOD and MPO in groups.

Groups	Number	SOD (U/mg protein)	MPO (U/g wet tissue)
Normal	10	674 ± 37.78	0.29 ± 0.04
Control	10	495.5 ± 49.97**	1.03 ± 0.2**
Medilac-S	10	585.82 ± 95.67^##^	0.48 ± 0.16^##^

Note: **P < 0.01 compared with normal group; ^##^ *P* < 0.01 compared with control group.
